# Brief Considerations Relating to Convalescence

**Published:** 1905-07

**Authors:** E. E. Rowell

**Affiliations:** Stamford, Conn.


### Brief Considerations Relating to Convalescence.

By E. E. ROWELL, M. D., Stamford, Conn.

THE care of patients during the period of convalescence from acute diseases is a field where much more could be accomplished than is generally the case. At the beginning the attending physician exercises his best efforts in observation to make a correct diagnosis and, if accomplished, seeks to adopt a line of treatment which will avoid entirely or prevent the pitfalls which his experience tells him are possibly or probably in his patients' way. If, in spite of his care, the patient becomes progressively worse, or an unexpected serious crisis occurs, his activity necessarily redoubles, but once the crisis is passed and convalescence commences, it is too often the case that interest in the patient slackens and the doctor's treatment becomes more and more perfunctory and routine-like, everything being left to the recuperative powers of nature. In many cases this blind confidence may be justified by the outcome and the patient goes on to make a recovery. Too frequently, however, many patients are less fortunate and the tissue wastes caused by disease are slowly replaced or not at all, while the various organs of the body resume their interrupted functions only partially or imperfectly.

In convalescents, in whom nature takes up again the task of building up, not merely replacing tissue, the appetite is poor and the digestive process inadequate, so that the food usually taken in these cases is not correctly prepared for assimilation and nutrition. The mental status of a patient is abnormal; he takes no interest in his surroundings and there is no sharp rebound upwards, such as we may observe where proper nourishment is supplied from the first. The organs of elimination are also inactive and incompetent to perform their functions, increased as the demand is by the excessive oxidation and accumulation of waste products which attend at this stage in most diseased conditions. The nervous system also plays an important part in the establishment of a complete convalescence. Perfect performnace of its function by an organ depends upon the proper nutrition of the brain and spinal cord. Given a state of low vitality as in these cases of delayed convalescence, and all the organs of the body will suffer, whether their function be secretion or elimination, and their cells themselves, lack the requisite energy for taking up from the blood the materials necessary to their maintenance and to the development and formation of similar cells.

In all such cases, these several organs, the stomach and the intestines, the liver and the kidneys, the blood-making tissues, and finally, the brain and the spinal cord need a complete and perfect nutrition associated with mild stimulation. These wants I have found ideally combined in bovinine. It is a tonic and food par excellence and I have found it generally indicated in convalescence from most forms of acute disease.

---

Emergency Sutures.—If you have to tie a bloodvessel in an emergency, and you have no sterilised sutures, it will always be best to tie one end of the thread short and bring out the other end at one of the angles of the wound; allowing it to remain there. It will promote drainage, may be easily removed when it becomes loosened, and will diminish the chances of abscess formation.—*International Journal Surgery.*

